# Hemodynamics with mechanical circulatory support devices using a cardiogenic shock model

**DOI:** 10.1038/s41598-024-64721-1

**Published:** 2024-06-19

**Authors:** Kazuyuki Yahagi, Gohki Nishimura, Kei Kuramoto, Yusuke Tsuboko, Kiyotaka Iwasaki

**Affiliations:** 1 Cooperative Major in Advanced Biomedical Sciences, Joint Graduate School of Tokyo Women’s Medical University and Waseda University, Waseda University, 2-2 Wakamatsucho, Shinjuku, Tokyo 162-8480 Japan; 2 Division of Cardiology, Mitsui Memorial Hospital, Tokyo, Japan; 3 Department of Modern Mechanical Engineering, Graduate School of Creative Science and Engineering, Waseda University, Tokyo, Japan; 4 Waseda Research Institute for Science and Engineering, Waseda University, Tokyo, Japan; 5 Department of Integrative Bioscience and Biomedical Engineering, Graduate School of Advanced Science and Engineering, Waseda University, Tokyo, Japan; 6 Institute for Medical Regulatory Science, Comprehensive Research Organization, Waseda University, Shinjuku, Tokyo Japan

**Keywords:** Cardiogenic shock, Impella, Mechanical circulatory support, Veno-arterial extracorporeal membrane oxygenation, Cardiology, Preclinical research

## Abstract

Mechanical circulatory support (MCS) devices, including veno-arterial extracorporeal membrane oxygenation (VA-ECMO) and Impella, have been widely used for patients with cardiogenic shock (CS). However, hemodynamics with each device and combination therapy is not thoroughly understood. We aimed to elucidate the hemodynamics with MCS using a pulsatile flow model. Hemodynamics with Impella CP, VA-ECMO, and a combination of Impella CP and VA-ECMO were assessed based on the pressure and flow under support with each device and the pressure–volume loop of the ventricle model. The Impella CP device with CS status resulted in an increase in aortic pressure and a decrease in end-diastolic volume and end-diastolic pressure (EDP). VA-ECMO support resulted in increased afterload, leading to a significant increase in aortic pressure with an increase in end-systolic volume and EDP and decreasing venous reservoir pressure. The combination of Impella CP and VA-ECMO led to left ventricular unloading, regardless of increase in afterload. Hemodynamic support with Impella and VA-ECMO should be a promising combination for patients with severe CS.

## Introduction

Cardiogenic shock (CS) remains a clinical challenge associated with high mortality rates (1,2)^1,2^. Mechanical circulatory support (MCS) devices have been widely used for patients with CS, including those with advanced heart failure secondary to ischemic heart disease, cardiomyopathy, or myocarditis^1,2^. Previously, intra-aortic balloon pumping (IABP) and veno-arterial extracorporeal membrane oxygenation (VA-ECMO) were the mainstay for the management of CS^3–5^. In 2012, Thiele et al. reported the results of the IABP-SHOCK II trial, which failed to show that mechanical support with IABP improved the outcomes of patients with acute myocardial infarction (AMI) with CS^6^. Moreover, there was no difference in all-cause mortality between IABP and control groups at the 6-year long-term follow-up^7^. Based on this randomized clinical trial, the current guidelines do not recommend the routine use of IABP therapy for CS^4,8^. VA-ECMO is a rescue therapy that involves powerful hemodynamic support for patients with severe CS. However, the disadvantage of VA-ECMO is an elevated left ventricular (LV) afterload, resulting in greater LV workload. Impella, a catheter with a small built-in axial flow pump (Abiomed, Danvers, MA, USA), has been approved for use in CS. Impella is expected to have an unloading effect because it can pump blood directly from the left ventricle^9^. Additionally, the combination of Impella and VA-ECMO improves hemodynamics and mortality^10,11^. However, the mortality rate of patients with refractory shock is extremely high^12^. Hemodynamics with Impella or the combination of Impella and VA-ECMO has not been thoroughly understood.

Assessment of hemodynamics, including pressure and flow in the aorta and venous system, and ventricular function, which is represented using a pressure–volume loop during a complete cardiac cycle, is valuable for understanding MCS^13,14^. Although an in vitro circulation study is a powerful tool for understanding hemodynamics in a specific target^15,16^, there has been no comprehensive study on hemodynamics with MCS. Here, we assessed the effects of Impella, VA-ECMO, and their combined use on hemodynamic changes using a clinically relevant circulatory system simulating CS.

## Methods

### CS model with a pulsatile flow system

**Figure 1:**
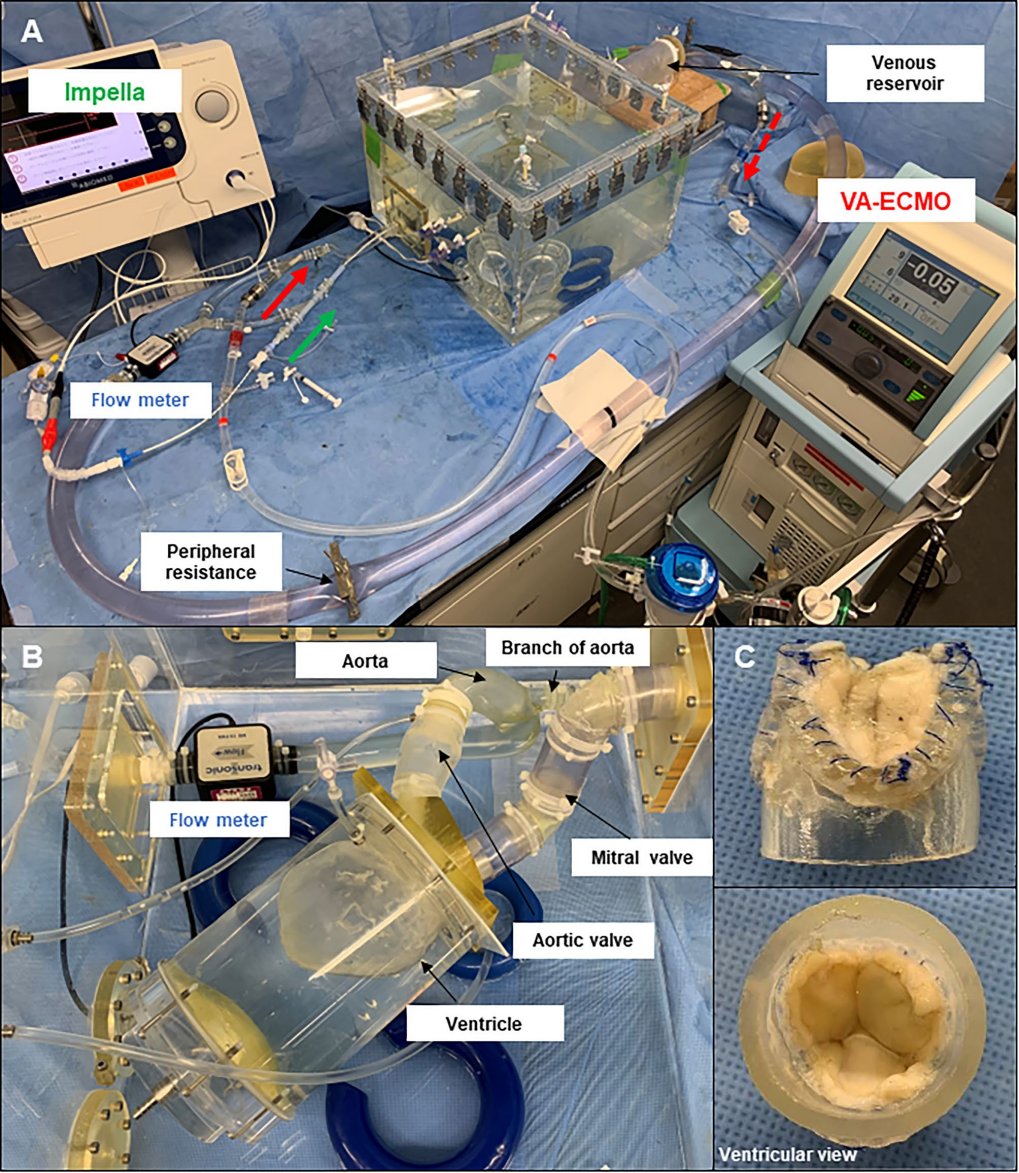
Experimental setup. (**A**) A pulsatile circulation model with a venous system; (**B**) A ventricular model with an aorta and a branch (brachiocephalic artery), an aortic valve, and a mitral valve; (**C**) An aortic valve made of bovine pericardium.

The pulsatile circulatory system consisted of a ventricular model, a mitral valve, an aortic valve, an elastic artery model, a systemic vascular resistance, and a venous reservoir with an elastic vessel model, which was modified based on our previous circulatory system (Fig. 1A,B)^16^. The silicone ventricular model was driven using a pneumatic console (VCT-50 (χ), Nipro, Osaka, Japan). The target blood pressure was achieved by adjusting the positive and negative pressures of the pneumatic console to 110 mmHg and − 5 mmHg, respectively. The aortic valve model comprised a three-dimensional (3D)-printed polymer frame with a tri-leaflet bovine pericardial tissue cusp (Fig. 1C). The mitral valve model comprised a 3D-printed polymer frame with a bi-leaflet polyurethane valve^17^. The silicone-composed artery model included a thoracic aorta with a brachiocephalic artery branch and iliac artery. Firstly, we designed a thoracic aortic model with a branch of brachiocephalic artery and an iliac artery, using computer-aided design (CAD) software (Solidworks 2019, Dassault Systemes Solidworks Corporation, Paris, France). The CAD data were then exported to a 3D printer (Eden260vs, Stratasys, Valencia, CA, USA). Secondly, each rigid artery model was constructed using a stereolithography resin (FullCure720, Altech, Tokyo, Japan). Thirdly, wax models of identical shape were constructed using a casting technique. Fourthly, the aortic models were fabricated from the wax molds using silicone (Shin-Etsu Silicone [KE-1603-A: KE-1603-B: KF-96-50CS; 5:5:3], Shin-Etsu Chemical, Tokyo, Japan). The stiffness parameter β of the aortic model was adjusted to 6.0 ± 0.1, equivalent to that of porcine aorta we obtained. The stiffness parameter β is the dimensionless inherent stiffness of blood vessels^18^. The dimensions of these models were determined based on human data^19–22^. Digital Imaging and Communications in Medicine images were imported into segmentation software (Mimics, Materialize, Leuven, Belgium), and the 3D models were reconstructed. The total volume capacity of the circulation loop was approximately 5500 ml, including 500 ml in the ventricle (270 ml) with conduit, 1500 ml on the arterial side, and 3500 ml on the venous side. The mean circulatory filling pressure was controlled by the volume of physiological saline solution and was set at 7 mmHg for the normal condition and 13 mmHg for the CS condition.

### Assessment of hemodynamics

Heart rate was maintained at 75 beats/min at a fixed rate. Pressure was measured in the ventricle, aorta, and venous reservoir using pressure transducers (PX600 TruWave; Edwards Lifesciences, Irvine, CA, USA). The flow was measured at the aorta, distal artery (iliac artery), and branch of the aorta (brachiocephalic artery) using an ultrasonic flow sensor (ME-PXN ME19PXN325; Transonic, Ithaca, NY, USA). The flow sensor in the distal artery was located at the distal part of the cannulation site for the VA-ECMO outlet (Fig. 1A). The mean total flow was calculated as the mean flow at the distal artery (iliac artery) and brachiocephalic artery. Aortic pressure was adjusted by peripheral resistance with a tube clamp. Peripheral resistance in the CS condition was the same as that in the normal condition. The brachiocephalic artery flow was adjusted using a tube clamp. Previous reports have shown that normal cerebral blood flow represents approximately 14% of the total cardiac output, which reduces in heart failure^23^. A conductance catheter (CD Leycom, Zoetermeer, The Netherlands) was placed in the LV, and the pressure–volume loop of the LV, including mean peak ventricular pressure (VP), mean end-diastolic pressure (EDP), mean end-diastolic volume (EDV), and mean end-systolic volume (ESV), was assessed. Mean stroke volume (SV) was calculated as the difference between EDV and ESV. These parameters were measured under each set of experimental conditions.

### CS model with MCS devices

Hemodynamics was assessed based on the CS status using MCS devices, including Impella CP and VA-ECMO (Senko Medical Instrument, Tokyo, Japan). Impella CP can deliver up to a mean maximum of 3.7 L per minute (L/min) from the ventricle into the aorta. Impella CP has 10 performance levels ranging from P0 to P9 (0 to 46,000 revolutions per minute [rpm]). In the circulation system, Impella CP was placed from the right iliac artery to the ventricle.VA-ECMO drained from the venous reservoir and returned to the left iliac artery.

## Results

### Hemodynamics of normal and CS conditions

**Figure 2:**
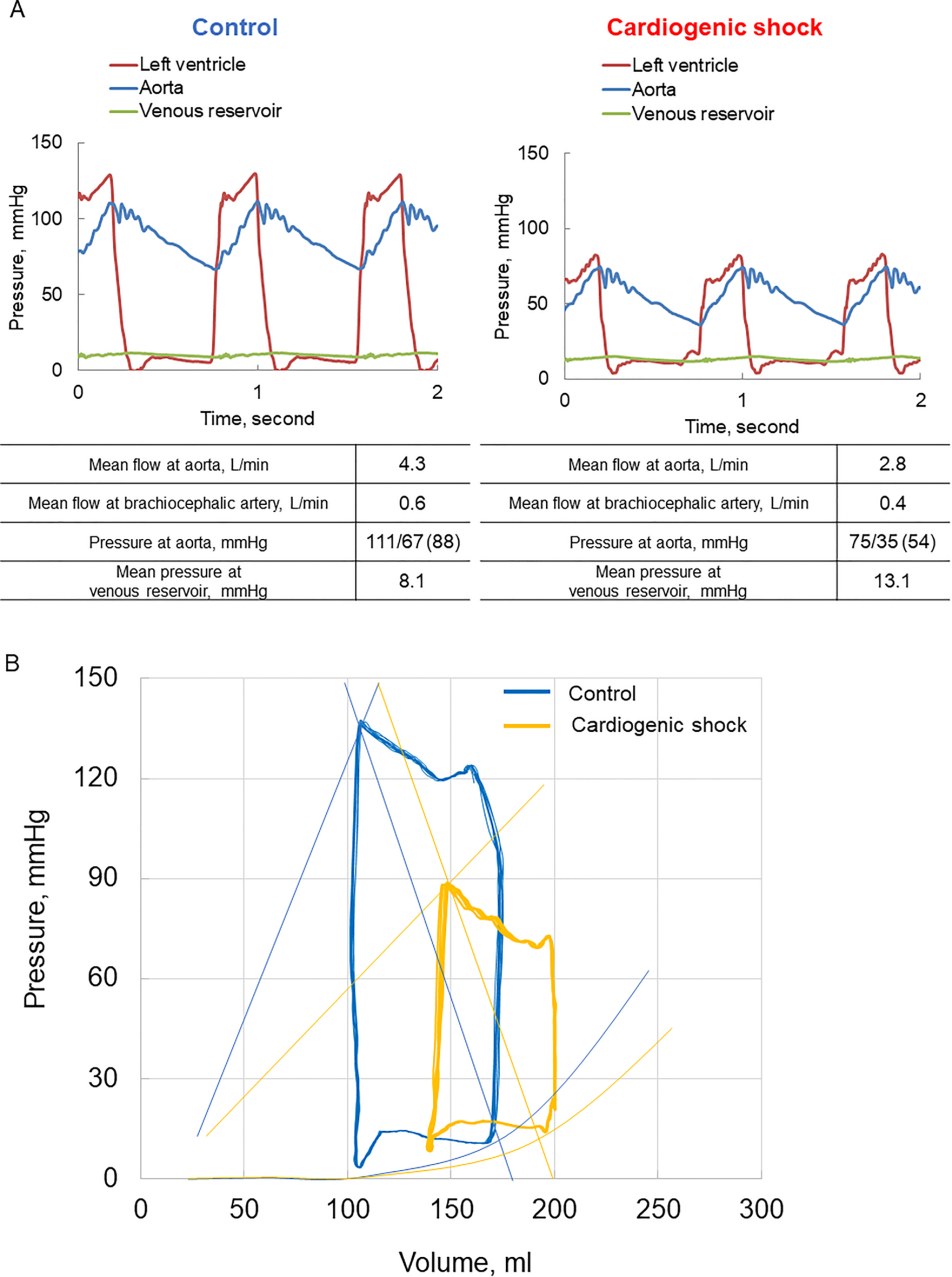
Representative hemodynamic waveform and pressure–volume loop. Representative hemodynamic waveform (**A**) and pressure–volume loop (**B**) of the left ventricle of control and cardiogenic shock model. Blue line, control; Yellow line, cardiogenic shock.

Under normal conditions, aortic pressure was 111/67 (88) mmHg, mean aortic flow was 4.3 L/min, mean flow at the brachiocephalic artery was 0.6 L/min (12.2% of total cardiac output), and mean venous reservoir pressure was 8.1 mmHg (Fig. 2A). Regarding the CS status, aortic pressure was 75/35 (54) mmHg, mean aortic flow was 2.8 L/min, mean flow at the brachiocephalic artery was 0.4 L/min (12.5% of total cardiac output), and mean venous reservoir pressure was 13.1 mmHg. The pressure–volume loop is shown in Fig. 2B. Under normal conditions, the peak VP, EDP, ESV, EDV, and SV were 137 mmHg, 13 mmHg, 107 ml, 169 ml, and 62 ml, respectively. Under CS conditions, a lower peak VP (88 mmHg), higher EDP (24 mmHg), higher ESV and EDV (148 and 199 ml, respectively), and smaller SV (51 ml) were observed compared with those under normal conditions. Under CS conditions, a decrease in the end-systolic LV elastance (Ees) resulting from a decrease in contractility and the effective arterial elastance (Ea) moved to the right side due to increased preload (increased EDV) compared with those under normal conditions, which are well-known changes for low cardiac output (13). The pressure–volume loop was reproduced in accordance with a previous report (13). Using a clinically relevant CS model, we further assessed the influence of MCS on hemodynamics.

### Changes in CS hemodynamics by MCS

**Figure 3:**
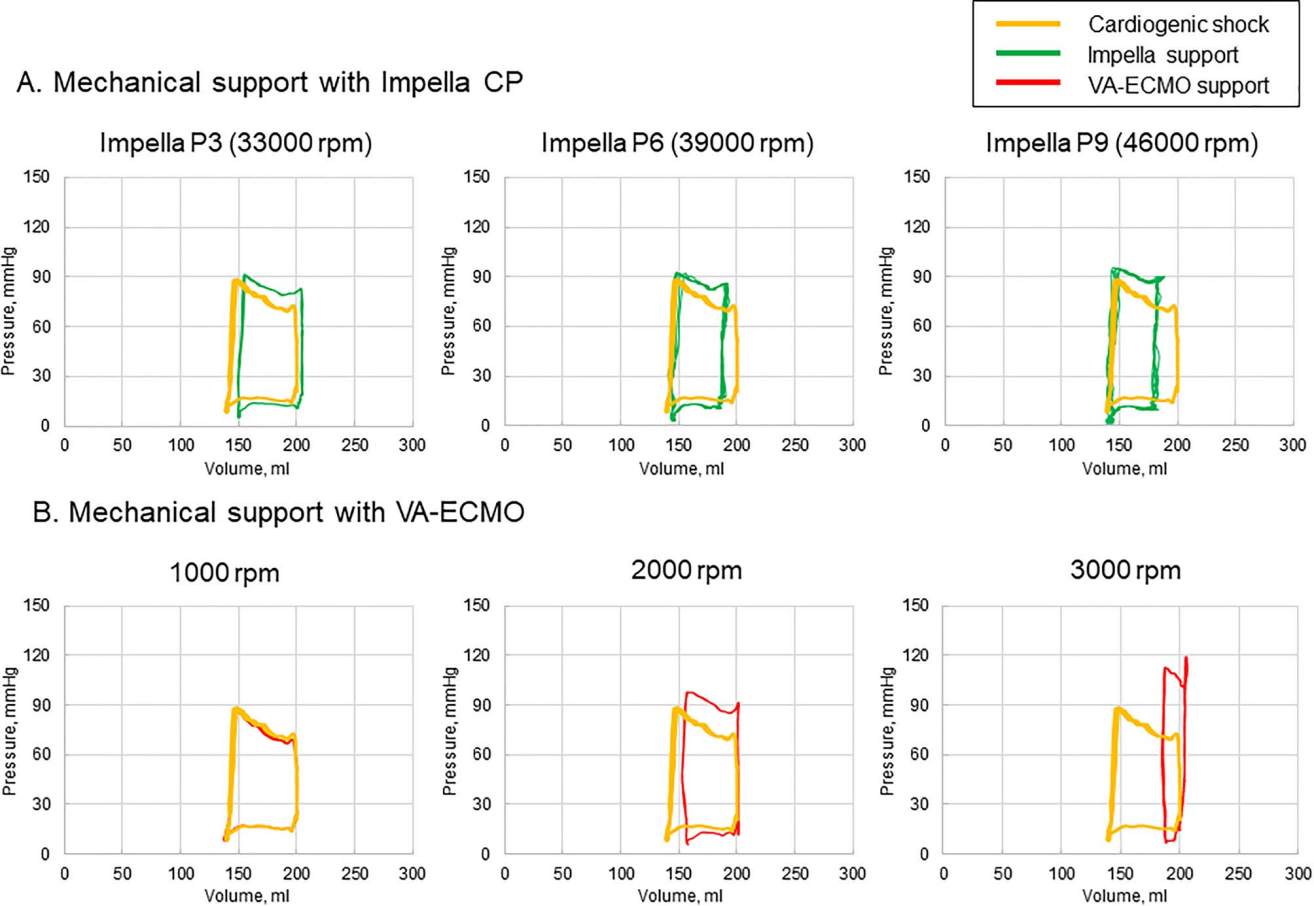
Representative pressure–volume loop of the left ventricle in the cardiogenic shock model. (**A**) Mechanical support with Impella CP at P3 (33,000 rpm), P6 (39,000 rpm), and P9 (46,000 rpm); (**B**) Mechanical support with veno-arterial extracorporeal membrane oxygenation (VA-ECMO) at 1000, 2000, and 3000 rpm. Yellow line: cardiogenic shock; green line: Impella support; red line: VA-ECMO support.

The flow level of Impella CP increased stepwise from P1 (23,000 rpm) to P9 (46,000 rpm), leading to increased mean total flow and mean aortic pressure (mean total flow: 2.70 L/min at P0 to 4.18 L/min at P9, mean pressure at the aorta: 46 mmHg at P0 to 76 mmHg at P9, respectively) (Table 1). Venous reservoir pressure decreased according to increased Impella flow (15.0 mmHg at P0 to 9.1 mmHg at P9). Based on the assessment of the pressure–volume loop, EDP/EDV decreased with increasing Impella flow level (Table 1, Fig. 3A). Consequently, SV decreased by increasing Impella flow, which was driven by reduction in EDV.

**Table 1:** Hemodynamics with Impella CP support.

	Impella CP									
	P0 0 rpm	P1 23 000 rpm	P2 31 000 rpm	P3 33 000 rpm	P4 35 000 rpm	P5 37 000 rpm	P6 39 000 rpm	P7 42 000 rpm	P8 44 000 rpm	P9 46 000 rpm
Mean total flow, L/min	2.70	3.27	3.64	3.70	3.82	3.89	3.96	4.07	4.14	4.18
Pressure at A, s/d (m), mmHg	73/23 (46)	78/39 (56)	82/48 (64)	83/52 (66)	84/55 (68)	85/57 (69)	86/59 (71)	88/61 (73)	90/63 (74)	91/65 (76)
Mean pressure at VR, mmHg	15.0	12.3	11.3	10.9	10.5	10.4	10.0	9.4	9.3	9.1
Peak LVP, mmHg	88	87	90	91	92	92	92	94	95	95
EDP, mmHg	24	21	20	20	18	18	18	17	18	17
ESV, ml	148	157	155	156	156	154	150	147	147	148
EDV, ml	199	210	204	199	203	193	188	187	184	180
Stroke volume, ml	51	53	50	42	47	39	38	40	37	32

*Mean total flow* mean flow of the iliac artery plus that of brachiocephalic trunk, *A* aorta, *EDP* end-diastolic pressure, *EDV* end-diastolic volume, *ESV* end-systolic volume, *VR* venous reservoir, *LVP* left ventricular pressure, *s* systolic, *d* diastolic, *m* mean.

The flow level of VA-ECMO increased stepwise to 1000, 1500, 2000, 2500, and 3000 rpm, leading to increased mean total flow and mean aortic pressure (mean total flow: 2.70 L/min at 0 rpm to 4.47 L/min at 3000 rpm, mean pressure at the aorta: 46 mmHg at 0 rpm to 97 mmHg at 3000 rpm, respectively) (Table 2, Fig. 3B). Venous reservoir pressure decreased according to increased VA-ECMO support (15.0 mmHg at 0 rpm to 7.0 mmHg at 3000 rpm). The pressure–volume loop became increasingly narrow (decreased SV) and taller (increased afterload), leading to an increased pressure–volume area (myocardial oxygen demand) with increasing VA-ECMO support. SV decreased with increasing VA-ECMO support, driven by an increase in ESV. We consider that the effect of ventricular afterload may reduce the flow of Impella.

**Table 2:** Hemodynamic parameters with VA-ECMO support.

	VA-ECMO					
	0 rpm	1 000 rpm	1 500 rpm	2 000 rpm	2 500 rpm	3 000 rpm
Mean total flow, L/min	2.70	2.99	3.55	3.99	4.26	4.47
Pressure at A, s/d (m), mmHg	73/23 (46)	73/31 (51)	80/46 (62)	88/61 (74)	97/76 (85)	106/91 (97)
Mean pressure at VR, mmHg	15.0	13.1	11.1	9.7	8.1	7.0
Peak LVP, mmHg	88	86	92	98	105	112
EDP, mmHg	24	24	27	31	35	51
ESV, ml	148	148	152	200	171	189
EDV, ml	199	200	201	201	202	204
Stroke volume, ml	51	52	46	42	31	15

*Mean total flow* mean flow of the iliac artery plus that of brachiocephalic trunk, *A* aorta, *EDP* end-diastolic pressure, *EDV* end-diastolic volume, *ESV* end-systolic volume, *VR* venous reservoir, *LVP* left ventricular pressure, *s* systolic, *d* diastolic, *m* mean.

**Figure 4:**
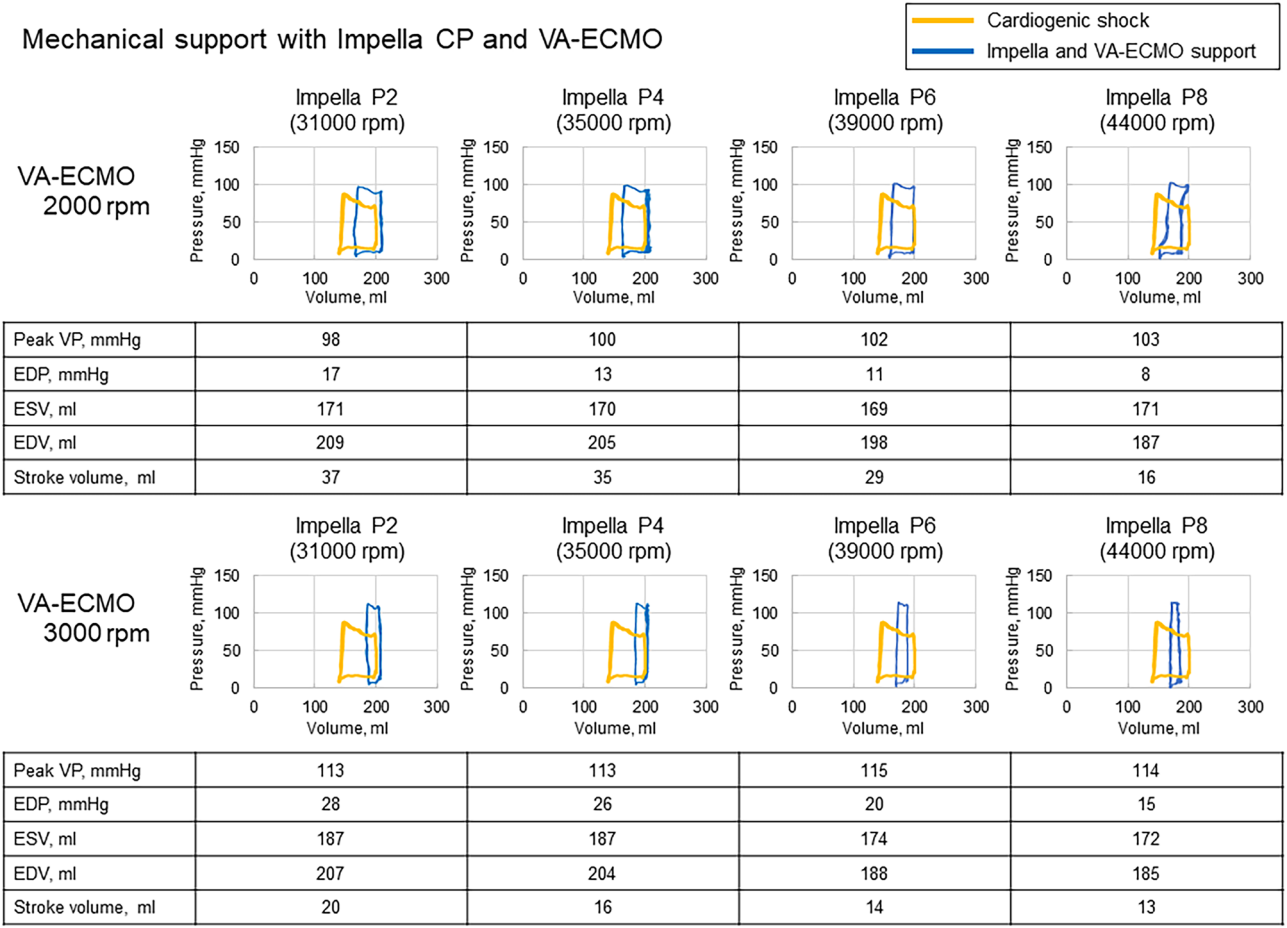
Representative pressure–volume loop of the left ventricle in the cardiogenic shock model with Impella CP and veno-arterial extracorporeal membrane oxygenation (VA-ECMO). *EDP* end-diastolic pressure, *EDV* end-diastolic volume, *ESV* end-systolic volume, *LVP* left ventricular pressure. Yellow line, cardiogenic shock; Blue line, Impella and VA-ECMO support.

Hemodynamics of the combination with Impella and VA-ECMO is shown in Table 3, and representative pressure–volume loops are shown in Fig. 4. The mean total flow and mean aortic pressure increased, and the mean venous reservoir pressure decreased by increasing either Impella CP or VA-ECMO support. The combination of Impella CP (P9, 46,000 rpm) and VA-ECMO (3000 rpm) showed maximum mean total flow (5.24 L/min), maximum mean arterial pressure (116 mmHg), and minimum venous reservoir pressure (5 mmHg). EDP decreased with increasing Impella flow level, even with VA-ECMO support. However, EDP was higher with high VA-ECMO support (3000 rpm) than with low VA-ECMO support (2000 rpm) due to increased afterload by VA-ECMO, even under Impella support. For example, EDP was higher with the combination of Impella CP at P9 and VA-ECMO at 3000 rpm than with Impella CP at P9 and VA-ECMO at 2000 rpm (15 mmHg vs. 8 mmHg).

**Table 3:** Hemodynamics with a combination of Impella CP and VA-ECMO support.

			Impella CP				
			P0 0 rpm	P2 31 000 rpm	P4 35 000 rpm	P6 39 000 rpm	P8 44 000 rpm
VA-ECMO	0 rpm	Mean total flow, L/min	2.70	3.64	3.82	3.96	4.14
		Pressure at A, s/d (m), mmHg	73/23 (46)	82/48 (64)	84/55 (68)	86/59 (71)	90/63 (74)
		Mean pressure at VR, mmHg	15.0	11.3	10.5	10.0	9.3
	2 000 rpm	Mean total flow, L/min	3.99	4.26	4.37	4.54	4.71
		Pressure at A, s/d (m), mmHg	88/61 (74)	92/69 (79)	93/72 (81)	96/79 (86)	98/83 (89)
		Mean pressure at VR, mmHg	9.7	8.1	8.3	7.6	7.3
	3 000 rpm	Mean total flow, L/min	4.47	4.69	4.69	4.78	5.24
		Pressure at A, s/d (m), mmHg	106/91 (97)	108/96 (101)	109/97 (102)	110/101 (105)	120/114 (116)
		Mean pressure at VR, mmHg	7.0	6.1	6.3	6.0	5.0

*A* aorta, *VR* venous reservoir, *s* systolic, *d* diastolic, *m* mean.

The results of the flow at the aorta, iliac arteries, and brachiocephalic artery are presented in Table 4. The mean total flow increased by increasing either the Impella or VA-ECMO support level. The mean aortic flow increased with Impella flow, but decreased with increasing VA-ECMO support. At 3000 rpm of VA-ECMO with Impella from P0 to P4, retrograde flow was observed in the aorta. However, antegrade flow in the aorta was observed at high Impella flow levels (P6 and P8). Importantly, there was a discrepancy in the flow between the aorta and distal artery (iliac arteries). The balance of the support level between Impella and VA-ECMO induces the discrepancy because Impella generates antegrade flow, whereas VA-ECMO generates retrograde flow. The mean flow of the brachiocephalic artery increased by increasing either the Impella or VA-ECMO support level, irrespective of the direction of the aortic flow.

**Table 4:** Mean flow in the aorta, iliac artery, and brachiocephalic trunk.

			Impella CP				
			P0 0 rpm	P2 31 000 rpm	P4 35 000 rpm	P6 39 000 rpm	P8 44 000 rpm
VA-ECMO	0 rpm	Aorta, L/min	2.28	3.03	3.21	3.34	3.47
		Iliac artery, L/min	2.33	3.14	3.30	3.41	3.57
		Brachiocephalic artery, L/min	0.38	0.50	0.53	0.55	0.57
		Total, L/min	2.70	3.64	3.82	3.96	4.14
	2 000 rpm	Aorta, L/min	1.41	1.63	1.82	2.16	2.37
		Iliac artery, L/min	3.47	3.67	3.77	3.92	4.05
		Brachiocephalic artery, L/min	0.52	0.59	0.60	0.62	0.67
		Total, L/min	3.99	4.26	4.37	4.54	4.72
	3 000 rpm	Aorta, L/min	− 0.27	− 0.05	− 0.10	0.07	0.60
		Iliac artery, L/min	3.85	4.00	4.00	4.08	4.46
		Brachiocephalic artery, L/min	0.63	0.69	0.69	0.70	0.77
		Total, L/min	4.47	4.69	4.69	4.78	5.24

*Total flow* flow of the iliac artery plus brachiocephalic artery.

## Discussion

**Figure 5:**
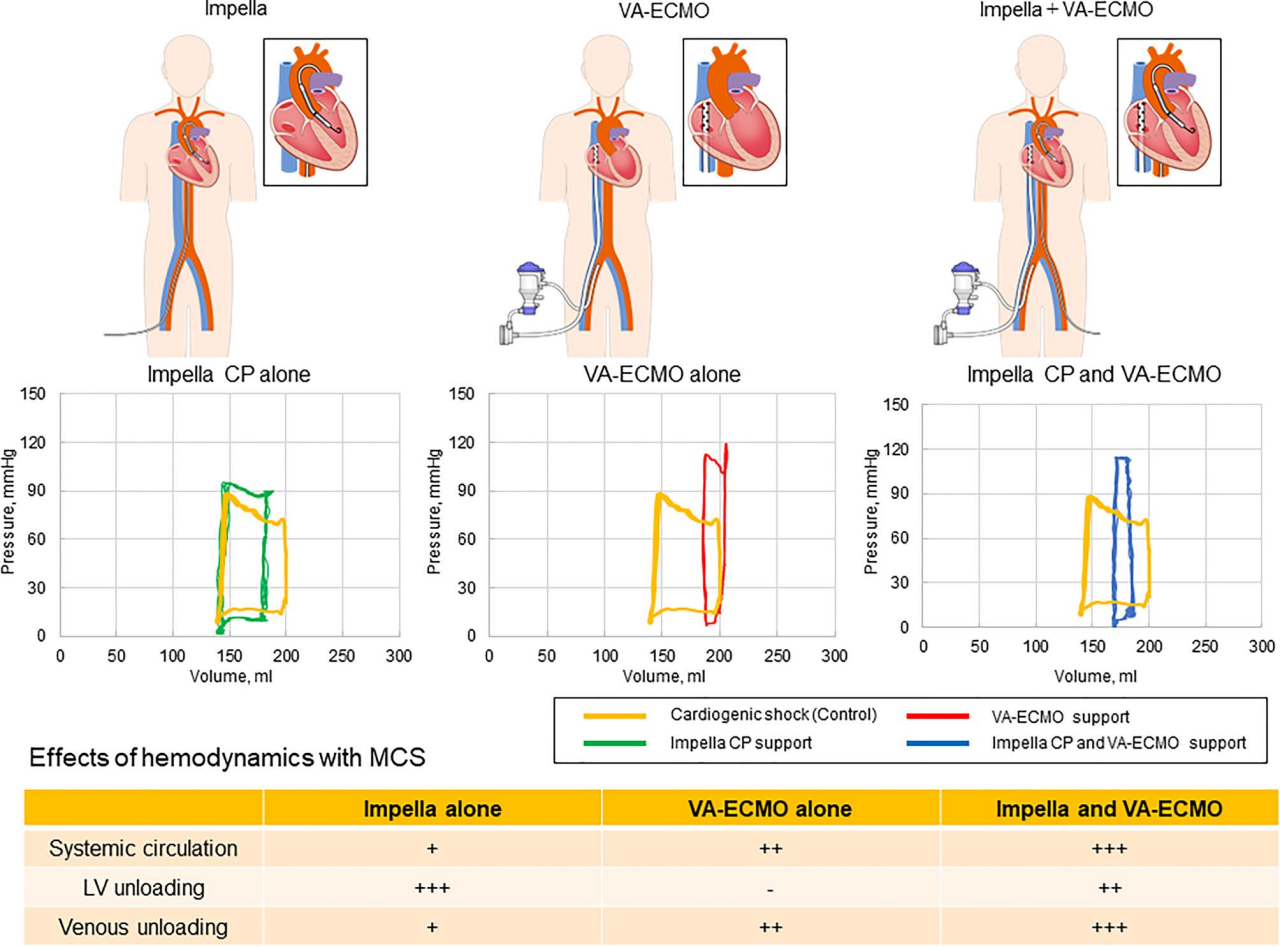
Representative pressure–volume loop of Impella alone, veno-arterial extracorporeal membrane oxygenation (VA-ECMO) alone, and combination of Impella and VA-ECMO and effects of hemodynamics with mechanical circulatory support (MCS).

We successfully created an experimental pulsatile flow system with venous function that can be used to assess MCS, including Impella and VA-ECMO. The main findings of the current study are as follows: (1) the Impella circulatory support device could increase forward flow and arterial pressure and decrease EDV and EDP (LV unloading); (2) VA-ECMO could increase arterial pressure owing to increased LV afterload and alter the direction of the aortic flow (antegrade or retrograde) depending on the flow level with VA-ECMO; (3) VA-ECMO could significantly reduce venous pressure (venous unloading) compared with Impella CP; and (4) the combination of Impella and VA-ECMO could lead to the greatest hemodynamic support with LV unloading (Fig. 5).

Clinical evidence, including high-quality clinical trials such as randomized controlled studies for patients with CS, is scarce because of the difficulties inherent in these challenging populations. Therefore, in vitro experimental and animal studies are valuable for understanding the hemodynamics of MCS in this field. Several animal studies have been conducted to assess the hemodynamics with Impella^24,25^. However, it is difficult to perform detailed assessments even in animal studies. The pulsatile flow circulation system in this study was created based on our previous model^16^ and expanded to assess hemodynamics with clinically available MCS devices.

Several preclinical and clinical studies have demonstrated the advantage of the Impella circulatory support device for high-risk coronary intervention or CS, resulting from increased blood flow and LV unloading^9,26^. Similar to previous reports, this study showed ventricular unloading with Impella, and significant unloading was observed with increased Impella flow.

The benefit of VA-ECMO is an increase in aortic pressure and a decrease in venous pressure (venous unloading). However, an increase in EDP could be a problem for early heart recovery, especially after AMI. Increased blood pressure and EDP are associated with a greater pressure–volume area, resulting in greater myocardial oxygen demand, which prevents myocardial healing^27^. This study indicated that the effect of decreasing venous pressure (venous unloading) was greater with VA-ECMO than with Impella CP. Previous reports have shown that elevated central venous pressure resulting in worsening renal function leads to cardiovascular events^28,29^. Therefore, VA-ECMO should be an option to maintain hemodynamics in patients with severe CS who have high central venous pressure.

Although the combination of Impella and VA-ECMO showed better clinical outcomes than VA-ECMO alone^10,11^, the hemodynamics with the combination therapy remains unknown. In the current study, the combination of Impella and VA-ECMO resulted in the highest hemodynamic support (greatest total flow and arterial pressure). The settings of the flow levels in patients on Impella and VA-ECMO are extremely important for patient survival. In the severe condition of AMI, VA-ECMO might be needed for hemodynamic stability, although VA-ECMO results in delayed heart recovery due to increased LV afterload. Clinical studies including patients with AMI suggested that the incidence of death and rehospitalization with a smaller infarct myocardium was lower than that with a larger infarct myocardium^30^. In an animal study, strong Impella support led to smaller myocardial infarction than no support^25^. Therefore, increased Impella support leading to lower EDP/EDV should be considered for the recovery of cardiac function once hemodynamics stabilizes.

Most recently, a large registry including patients with CS from the Mayo Clinic showed that the mortality in patients with refractory shock was extremely high^12^. Moreover, hypoperfusion leads to greater mortality than hypotension^31^. Therefore, early hemodynamic support with sufficient cardiac output is imperative to increase the survival rate. Clinically, insufficient support with Impella due to suction was observed in some cases, even in cases of volume overload due to cardiac failure. Possible reasons for suction were low volume, incorrect pump position, and inadequate LV filling due to right ventricular failure. One of the issues in patients with acute heart failure is hypoxic pulmonary vasoconstriction^32^. When Impella support is insufficient, escalation with VA-ECMO or a left ventricular assist device (LVAD) should be considered. Additionally, in cases with precapillary pulmonary hypertension, decreasing pulmonary resistance with a pulmonary vasodilator, such as a phosphodiesterase III inhibitor and inhaled nitric oxide, may be another option to increase cardiac output^33^.

North–south syndrome is one of the complications after the implantation of VA-ECMO. Forward flow was observed even in VA-ECMO support when the flow from the proximal aorta was greater than that from the distal aorta, and increased flow with VA-ECMO resulted in retrograde flow at the aorta. The present study showed that retrograde aortic flow was observed when VA-ECMO support was strong (3000 rpm). In addition, flow stagnation might be present between P4 (35,000 rpm) and P6 (39,000 rpm) with Impella CP under VA-ECMO (3000 rpm), because the flow of the aorta was almost zero. A recent case report described the presence of a large mobile thrombus in the descending aorta in a patient with CS supported by a combination of Impella (2.0 L/min) and VA-ECMO (3.0 L/min)^34^. Theoretically, when the retrograde flow of VA-ECMO is equivalent to the forward flow, the aortic flow is likely to stagnate. From the present study and case report, both Impella and VA-ECMO with high-flow support in patients with CS might be at risk of inducing thrombus formation because of the stagnation of the aortic flow, although the situation should be different based on cardiac function.

Currently, MCS devices are widely used in patients with CS. However, obtaining strong evidence in this field is difficult. For example, the recommendation of IABP use has been downgraded, and there has been no randomized controlled study of VA-ECMO. Thus, in vitro clinically relevant studies with circulation models are imperative for assessing the influence of MCS on hemodynamics in CS patient analogs with various settings and answering clinical questions in real-world clinical practice. We believe that the present circulation system should be useful for hemodynamic assessment, even for other MCS.

This study has several limitations. First, the present study was conducted using an in vitro circuit without the right ventricle. Therefore, the hemodynamics of the right heart could not be assessed. Second, the present study used a relatively large size of the ventricle to maintain circulation system. Third, we assessed only one setting of CS status using Impella and VA-ECMO. Clinical situations include many different conditions; therefore, the data from the present study may not be widely applied to clinical situations. However, the trend of MCS effects can be understood. Fourth, the present study suggests that the combined use of Impella and VA-ECMO might be the best strategy with strong hemodynamic support for patients with severe CS, but higher incidence of complications, including cannula-site bleeding, following Impella implantation has been reported than with IABP. Therefore, careful assessment of the bleeding risk is required for the treatment with MCS.

## Conclusions

Left ventricular unloading is a specific advantage of the Impella circulatory support system that leads to improved hemodynamics. VA-ECMO is a powerful device to support hemodynamics with venous unloading, although an increased cardiac load was induced. The combination of Impella and VA-ECMO should be a valuable option for patients with severe CS. We believe that the findings using the present pulsatile circulation system may provide valuable insights into optimizing the MCS strategy for the management of patients with CS.

## Data Availability

The data that support the findings of this study are available from the corresponding author upon reasonable request.

## Abbreviations

CS: Cardiogenic shock
MCS: Mechanical circulatory support
IABP: Intra-aortic balloon pumping
VA-ECMO: Veno-arterial extracorporeal membrane oxygenation
AMI: Acute myocardial infarction
LV: Left ventricular
Ees: End-systolic LV elastance
Ea: Arterial elastance
LVAD: Left ventricular assist device
